# A fungal phylogeny based on 82 complete genomes using the composition vector method

**DOI:** 10.1186/1471-2148-9-195

**Published:** 2009-08-10

**Authors:** Hao Wang, Zhao Xu, Lei Gao, Bailin Hao

**Affiliations:** 1 T-life Research Center, Department of Physics, Fudan University, Shanghai 200433, PR China; 2 Department of Botany & Plant Sciences, University of California, Riverside, CA(92521), USA; 3 Institute of Theoretical Physics, Academia Sinica, Beijing 100190, PR China; 4 Santa Fe Institute, Santa Fe, NM(87501), USA

## Abstract

**Background:**

Molecular phylogenetics and phylogenomics have greatly revised and enriched the fungal systematics in the last two decades. Most of the analyses have been performed by comparing single or multiple orthologous gene regions. Sequence alignment has always been an essential element in tree construction. These alignment-based methods (to be called the standard methods hereafter) need independent verification in order to put the fungal Tree of Life (TOL) on a secure footing. The ever-increasing number of sequenced fungal genomes and the recent success of our newly proposed alignment-free composition vector tree (CVTree, see Methods) approach have made the verification feasible.

**Results:**

In all, 82 fungal genomes covering 5 phyla were obtained from the relevant genome sequencing centers. An unscaled phylogenetic tree with 3 outgroup species was constructed by using the CVTree method. Overall, the resultant phylogeny infers all major groups in accordance with standard methods. Furthermore, the CVTree provides information on the placement of several currently unsettled groups. Within the sub-phylum Pezizomycotina, our phylogeny places the Dothideomycetes and Eurotiomycetes as sister taxa. Within the Sordariomycetes, it infers that *Magnaporthe grisea *and the Plectosphaerellaceae are closely related to the Sordariales and Hypocreales, respectively. Within the Eurotiales, it supports that *Aspergillus nidulans *is the early-branching species among the 8 aspergilli. Within the Onygenales, it groups *Histoplasma *and *Paracoccidioides *together, supporting that the Ajellomycetaceae is a distinct clade from Onygenaceae. Within the sub-phylum Saccharomycotina, the CVTree clearly resolves two clades: (1) species that translate CTG as serine instead of leucine (the CTG clade) and (2) species that have undergone whole-genome duplication (the WGD clade). It places *Candida glabrata *at the base of the WGD clade.

**Conclusion:**

Using different input data and methodology, the CVTree approach is a good complement to the standard methods. The remarkable consistency between them has brought about more confidence to the current understanding of the fungal branch of TOL.

## Background

Fungi make up one of the major Eukaryotic kingdoms besides the Plantae and Animalia. These heterotrophic organisms possess a chitinous cell wall and grow as single cells or as multicellular mycelium made of hyphae. Although some species are not capable of forming specialized reproductive structures and propagate solely by vegetative growth, many fungi reproduce sexually and asexually via spores. To date, around 70 000 fungal species have been described while the total number of species has been estimated at 1.5 million [1,2].

Since the early 1990s, the introduction of molecular characters has drastically revised the traditional fungi phylogenetic system based on morphology, physiology and sexual states. Numerous works have addressed cladistic relationships among all major groups of the kingdom [2-5]. Molecular characters have shown great power when morphological characters are convergent, reduced, or missing among the taxa. So far most fungal molecular phylogenetic inferences have been established on alignment of single or several orthologous gene loci [3,6]. When multi-locus data are investigated, the commonly adopted methods are gene concatenation and consensus tree analysis [4]. Since more genes provide more phylogenetic information, many recent phylogenomic studies tried to infer phylogenies for various organisms by combining large datasets of aligned genes (or ESTs) [7-9]. For example, Robbertse *et al *[10] and Fitzpatrick *et al *[4] built phylogeny from large datasets of protein-coding genes of 17 and 42 genomes, respectively.

These methods have achieved great success in the last two decades. However, some well-documented stochastic or systematic errors in tree reconstruction often lead to incongruent results [11,12]. Furthermore, their applications depend on manual selection of many parameters and fine adjustments of sequence data. For example, at least at some stages, the standard methods select and process genes (and sites) to avoid systematic errors [11]. These problems broach a question of principle: the phylogeny based on sequence alignment needs an independent verification in order to put the fungal TOL on a more secure footing. Recently, methods based on other strategies such as gene content, gene order and the distribution of oligonucleotides or peptides have been proposed to infer phylogenies (see [12] and references therein), which have made the verification feasible.

We have constructed a kingdom-wide fungal phylogenetic tree for 82 sequenced genomes using an alignment-free composition vector (CV) method [13-16]. The method has previously been successfully applied to prokaryotic and viral phylogenies [16,17]. It uses whole-genome data of organisms and excludes artificial selection of genes and sites. In this report we will compare in detail our phylogenetic inferences with those inferred from standard methods. We will show the striking consistency between them and discuss the relationships among controversial lineages. Since our method reconstructs the fungal phylogeny with independent input data and methodology, the CVTree is a strong independent verification and complement to, but not a substitution for the traditional alignment-based analysis.

## Results and discussions

### Higher-level phylogeny

#### Basal splits and the Dikarya

**Figure 1:**
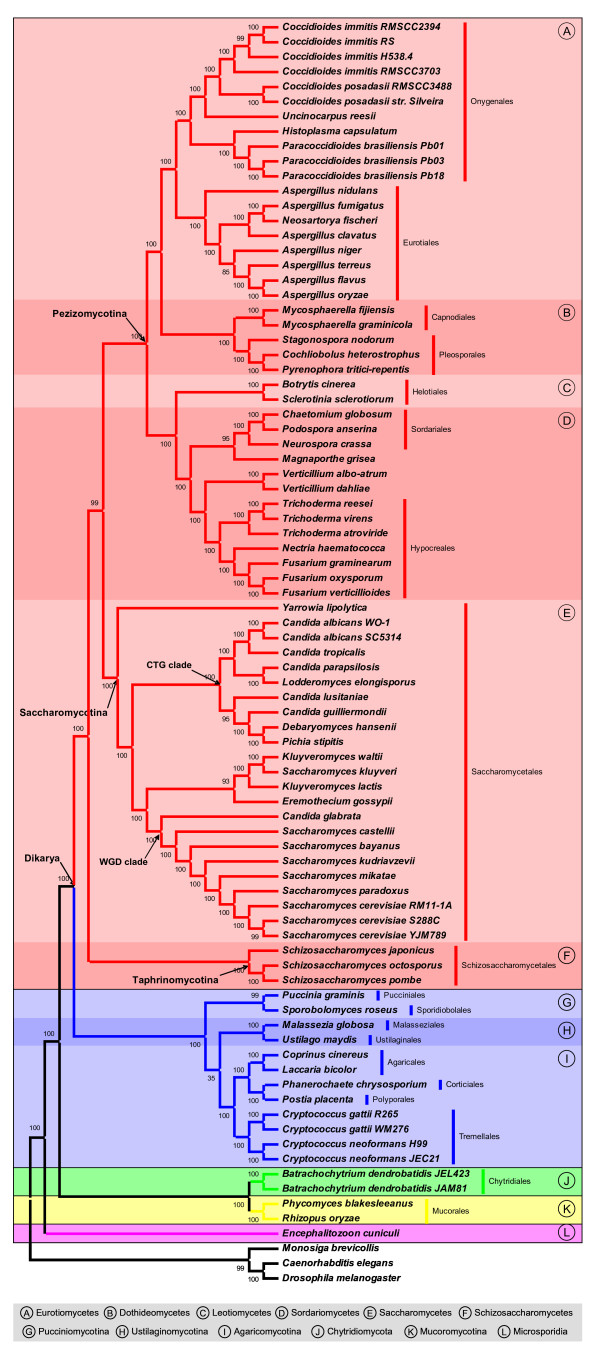
**The CVTree of 82 fungi**. The CVTree of 82 fungi. This tree is obtained with K = 7. Bootstrap values (100 bootstrap replicates; [see Additional file 3] and [13] for details) are reported as percentages. Strain names are given only when more than one organism in that species appeared in our dataset. Blocks colored in red, blue, green, yellow and purple correspond to the Ascomycota, Basidiomycota, Chytridiomycota, Mucoromycotina and Microsporidia, respectively. Major groups in the Ascomycota and Basidiomycota are distinguished by alternate red and blue colors, respectively.

Figure 1 represents the CVTree of the 82 sequenced fungi. The organisms are grouped into 5 phyla or subphyla at the highest level, with the Ascomycota and Basidiomycota forming a monophyletic group Dikarya. Because currently available genomes in Chytridiomycota (2 strains of the same species, *Batrachochytrium dendrobatidis*), Microsporidia (*Encephalitozoon cuniculi *only) and Mucoromycotina (2 genera of the same family, *Rhizopus oryzae *and *Phycomyces blakesleeanus*) lack diversity, it is inappropriate to fully discuss the relationships among these clades until more organisms are sequenced. The following discussion will focus on the Basidiomycota and Ascomycota.

#### The Basidiomycota

The phylum Basidiomycota consists of 3 subphyla: Agaricomycotina, Pucciniomycotina and Ustilaginomycotina. Except for *Malassezia globosa*, the 11 Basidiomycetes are classified into 5 classes, 7 orders, 7 families, 8 genera and 9 species in the scheme of the NCBI taxonomy. *M. globosa *is marked as Ustilaginomycotina *incertae sedis *in the NCBI taxonomy browser [18]. The CVTree places it as sister taxon to *Ustilago maydis *(Figure 1, block H). This topology is supported by recent analyses of rDNA data and concatenated single-copy orthologous proteins [19,20].

Although each of the three subphyla is widely accepted as monophyletic group, their relationships are not well-resolved [3]. Previous cytological, biochemical and molecular analyses [2,21] have suggested a topology like (Pucciniomycotina, (Agaricomycotina, Ustilaginomycotina)). With highly restricted taxon sampling in the Pucciniomycotina and Ustilaginomycotina, the CVTree recovers the same topology, but the bootstrap value is rather low (Figure 1, blocks in blue). Broader taxon sampling in each subphyla and further investigations are necessary to address this difficult question.

#### The Ascomycota

The 65 Ascomycetes come from three subphyla: the Pezizomycotina, Saccharomycotina and Taphrinomycotina. Although the monophyly of the Taphrinomycotina has not been fully agreed [22-25], the fission yeasts Schizosaccharomycetes (Taphrinomycotina) have been widely taken as a basal lineage of the Ascomycota [2,3,5,25]. Our results support the early divergence of *Schizosaccharomyces *and a close relationship between the Pezizomycotina and Saccharomycotina (Figure 1). In the current dataset, organisms from the Taphrinomycotina and Saccharomycotina come from only one order in either subphylum, so it is proper to focus our higher-than-order level discussion on the relationships within the subphylum Pezizomycotina, where we have 39 organisms distributed in 4 classes.

The CVTree recognizes 4 class-level clades within the Pezizomycotina: the Dothideomycetes, Eurotiomycetes, Leotiomycetes and Sordariomycetes. They all come from the well-supported "Leotiomyceta" clade but their relationships have not been well resolved [26]. Lutzoni *et al *[2] placed the Sordariomycetes and Dothideomycetes as sister clades in their four-gene analysis. However, subsequent works based on single- and multi-gene(s) found that it was the Leotiomycetes rather than Dothideomycetes that should be sister to the Sordariomycetes and they formed a clade [4,10,25,26]. Our CVTree confirms the latter topology (Figure 1).

Although many accept the close relationship between the Sordariomycetes and Leotiomycetes, the relationships among the Dothideomycetes, Eurotiomycetes and Sordariomycetes-Leotiomycetes clade were less clear. At least three hypotheses have been proposed: (1) the Dothideomycetes and Sordariomycetes-Leotiomycetes group together to the exclusion of Eurotiomycetes [26]. (2) The Eurotiomycetes and Sordariomycetes-Leotiomycetes group together to the exclusion of Dothideomycetes [4,10,25]. (3) The Dothideomycetes and the Eurotiomycetes form a clade that is sister group to the Sordariomycetes-Leotiomycetes clade [4,10]. By including in their analyses only one species (*Stagonospora nodorum*) of Dothideomycetes, both Fitzpatrick *et al *[4] and Robbertse *et al *[10] reported conflicting results: depending on the tree construction model used, their results supported either hypothesis (2) or (3). By contrast, the current work adds in four more species (i.e., *Cochliobolus heterostrophus*, *Pyrenophora tritici-repentis*, *Mycosphaerella fijiensis *and *Mycosphaerella graminicola*) and our result unambiguously places the Dothideomycetes sister to Eurotiomycetes, supporting hypothesis (3) (Figure 1, block A and B).

Within the Sordariomycetes, Hypocreales and Sordariales are two well-supported order-level clades. However, it is uncertain in which order *M. grisea *(family Magnaporthaceae) should be placed (e.g. both Index Fungorum [27] and NCBI taxonomy browser [18] categorize it as Sordariomycetes *incertae sedis*). The CVTree suggests that it is closely related to the Sordariales (Figure 1, block D). This placement concurs with some recent phylogenomic analyses [3,4,10,28]. Similarly, the order-level classification of two Verticillium species (family Plectosphaerellaceae) is not fully resolved either. Our tree places the Plectosphaerellaceae sister to the Hypocreales (Figure 1, block D), and this relationship is supported by the 4-gene phylogeny of Zhang *et al *[28] as well as Index Fungorum. Index Fungorum categorizes the family into the subclass Hypocreomycetidae, which includes in the Hypocreales. Regarding the other three classes, none of them have more than two ordinal members, so the order-level relationships within them could only be trivial.

### Lower-than-order-level phylogeny

Comparing to higher-level relationships, there have been more disagreements regarding the classification of taxa lower than order. This was partially caused by the difficulty to recognize various sexual states of one and the same species. Even the International Code for Botanic Nomenclature (Article 59) [29] permits to give anamorph a separate name from the corresponding teleomorph. However, molecular characters are capable to reveal more definite relationships.

In our dataset there are 7 orders (i.e., the Schizosaccharomycetales from the Taphrinomycotina; the Eurotiales, Hypocreales, Onygenales, Pleosporales and Sordariales from the Pezizomycotina; and the Saccharomycetales as the unique order in the Saccharomycotina) in which the number of sequenced organisms is greater than two. Within the Pleosporales, the relationships among the 3 organisms are less controversial: *P. tritici-repentis *and *C. heterostrophus *are from the family Pleosporaceae while *S. nodorum *is from the Phaeosphaeriaceae (Figure 1, block B). In what follows we discuss the phylogeny within the other 6 orders one by one.

#### The Schizosaccharomycetales

All the three organisms in this order belong to the genus *Schizosaccharomyces*. Before whole-genomes were available for members of the Schizosaccharomyces, their phylogenetic relationships were inferred from mitochondrial genomes [30]. Mitochondrial phylogeny shows that *Schizosaccharomyces pombe *and *Schizosaccharomyces octosporus *are more closely related to the exclusion of *Schizosaccharomyces japonicus*. The CVTree recovers the identical topology from whole-genome data (Figure 1, block F).

#### The Sordariales

In our analysis the order Sordariales is represented by 3 species: *Chaetomium globosum*, *Neurospora crassa *and *Podospora anserina*. According to the NCBI taxonomy they belong to the Chaetomiaceae, Sordariaceae and Lasiosphaeriaceae families, respectively. Previous analysis based on LSU rDNA (and other genes) have shown that many traditional families in this order do not form clades, e.g., the Chaetomiaceae and Lasiosphaeriaceae are paraphyletic groups [28,31,32]. Despite such inconsistency, recent 18S rDNA and phylogenomic analyses agreed that the relationships among the three species are ((*C. globosum*, *P. anserina*), *N. crassa*) [3,4,25,28,33]. Our CVTree phylogeny supports this topology as well (Figure 1, block D).

We mention in passing that so far the rice blast fungus *M. grisea *has been denoted as Sordariomycetes *incertae sedis*, i. e., not being designated to an existing order. From the CVTree and papers just cited above it comes out as ((Hypocreales, Plectosphaerellaceae), (*M. grisea*, Sordariales)), hinting on the feasibility of putting this species in a new order.

#### The Eurotiales

In the order Eurotiales all sequenced organisms are *Aspergillus *species, including *Neosartorya fischeri*, the teleomorph of *Aspergillus fischerianus*. The CVTree phylogeny (Figure 1, block A) of the 8 species is identical to the recent result of 30-gene phylogenomic analysis by Rokas *et al *(2007) [34] and to that shown in the BROAD-FIG Aspergillus Comparative Database [35]. In all of these trees *A. nidulans *is the basal lineage while in the previously widely accepted LSU rDNA phylogeny by Peterson (2000) [36] it did not ([see Additional file 1], Figure S1(a)). Another difference between Peterson's result and ours is the placement of *Aspergillus niger *and *Aspergillus terreus*: the former gave that *A. niger *was sister taxon to the *Aspergillus oryzae*-*Aspergillus flavus *clade and *A. terreus *diverged early. In contrast, our result supports the sister relationship of *A. terreus *and the *A. oryzae*-*A. flavus *clade and the early divergence of *A. niger*.

#### The Onygenales

In our dataset there are 4 species with 11 strains belonging to the Onygenales. The two *Coccidioides *species as well as *Uncinocarpus reesii *form one clade and the three *Paracoccidioides *as well as *Histoplasma capsulatum *form the other. Deeper in the tree, these two clades are sister taxa (Figure 1, block A). For a long time, *Paracoccidioides brasiliensis *was considered an imperfect fungus. In recent years, it has been considered a member of the family Onygenaceae and placed in a common group with *C. immitis*, *H. capsulatum *and *U. reesii *[37]. More recently, a clade distinct from Onygenaceae has been proposed as a new family Ajellomycetaceae to encompass *Histoplasma *and *Paracoccidioides *but not *C. immitis *and *U. reesii *[38,39]. The current work supports the suggestion of the Ajellomycetaceae by placing the Onygenaceae as its sister group.

#### The Hypocreales

In the Hypocreales and Saccharomycetales, taxonomy shows inadequate resolution and conflicts with current phylogeny derived from standard methods, i.e., some traditional families and genera turn out to be non-monophyletic. We discuss phylogeny of these two branches in the following two sections.

Although the molecular phylogenetic studies have helped in solving many problems that morphology could not, the classification of many members of Hypocreales, especially *Fusarium spp*. and *Trichoderma spp*. are far from being settled [40,41]. Regarding the 7 organisms in our dataset in the order, the key difference between the CVTree (Figure 1, block D) and NCBI taxonomy ([see Additional file 1], Figure S1(b)) is the position of *Fusarium oxysporum*. In the NCBI taxonomy, it is placed in the group mitosporic Hypocreales, while *Fusarium verticillioides *and *Fusarium graminearum *belong to the family Nectriaceae. In the CVTree, however, the three species form the Nectriaceae clade: *F. oxysporum *is grouped with *F. verticillioides *and *F. graminearum *is sister taxon to them. The monophyly of the clade is supported by Index Fungorum [27], which classifies *F. oxysporum *into the Nectriaceae. Moreover, the same topology can be found at BROAD-FGI Fusarium Comparative Database [42].

#### The Saccharomycetales

The Saccharomycetales is a unique order in the Saccharomycetes which in turn is a unique class in the subphylum Saccharomycotina according to Hibbett *et al *(2007) [5]. These species have been studied extensively and some members are model organisms. Two distinct events in their evolutionary history have been well-documented: (1) some species have undergone whole-genome duplication more than 100 million years ago. They form the so-called WGD clade; (2) some species translate CUG codon into serine instead of leucine. They form another branch called the CTG clade. Any reasonable phylogeny should clearly resolve the two clades among yeasts. The CVTree does.

Our current dataset includes 23 organisms from Saccharomycetales, as compared to 19 in Fitzpatrick *et al *(2006) [4] and 12 in James *et al *(2006) [3]. Therefore, we are in a position to perform a more detailed comparison of the CVTree with other phylogenies.

Within the Saccharomycetales, *Yarrowia lipolytica *is the early-diverging lineage and the other organisms consists of two groups covering the WGD and CTG clade, respectively (Figure 1, block E). This is a common feature of the 12-, 19 and 32-organism trees. Within the CTG clade, the CVTree gives a structure identical with that of Fitzpatrick *et al *[4] if *Pichia stipitis *is not included in. Our tree places *P. stipitis *and *D. hansenii *as sister taxa. This placement is consistent with the result of the 94 single-copy genes analysis [43], which suggests a close relationship between *D. hansenii *and *P. stipitis *to the exclusion of *C. lusitaniae *(Figure 1, block E). Our results further confirms two features in the CTG clade [4]: (1) *Candida guilliermondii *is closely related to *Debaryomyces hansenii *to the exclusion of *Candida lusitaniae *and (2) *Lodderomyces elongisporus *is closely related to *Candida parapsilosis *and is likely to be its sexual form.

The other group further splits into two clades by an ancient whole-genome duplication (WGD) event. The WGD clade includes six *Saccharomyces sensu stricto *species and two *Saccharomyces sensu lato *species (*Saccharomyces castellii *and *C. glabrata*). The ladderized topology within the *Saccharomyces sensu stricto *organisms is consistent with previous phylogenomic results [44-46]. So far the base of the WGD clade has not been confidently resolved. Some proposed that *S. castellii *diverged from the *Saccharomyces sensu stricto *species earlier than *C. glabrata *by comparing synteny among species and multi-gene analysis [4,47]. However, other phylogenomic analyses argued that *C. glabrata *diverged earlier [4,44,45]. According to the CVTree, *C. glabrata *is the likely basal lineage of the WGD clade (Figure 1, block E).

The monophyly of *Kluyveromyces waltii*, *Saccharomyces kluyveri*, *Eremothecium gossypii *and *Kluyveromyces lactis *is unsettled either. Some authors suggested that the four species are paraphyletic but they together with the WGD species constitute a clade [46,48]. For example, Kurtzman (2003) [46] proposed a topology like (((WGD, (*K. waltii*, *S. kluyveri*)), *K. lactis*), *E. gossypii*). In contrast, others proposed that the 4 species themselves form a clade [4,45]. The CVTree supports the monophyly of the four yeasts and the sister-relationship between this clade and the WGD group (Figure 1, block E).

The relationships among these 4 organisms are again controversial. Although many works agreed that (*K. waltii*, *S. kluyveri*) should be closer to each other, the grouping of *E. gossypii *and *K. lactis *are not widely accepted. Kurtzman [46] and Suh *et al *[48] proposed that they are paraphyletic, while Jeffroy *et al *[45] and Fitzpatrick *et al *[4] suggested that they form a clade and placed this group as sister branch to the (*K. waltii*, *S. kluyveri*) clade. The CVTree, unlike studies mentioned above, places *K. lactis *and (*K. waltii*, *S. kluyveri*) as sister group to the exclusion of *E. gossypii *(Figure 1, block E). As different materials and methods give controversial results, more genomes and analyses are required to confidently resolve this incongruence.

## Conclusion

To the best of sequenced fungi available, we have inferred their phylogeny using the alignment-free composition vector method and discussed their relationships. The above detailed comparison has shown remarkable consistency between the CVTree and the recent results of standard methods, the consistency actually holds at all levels. We can now give an overall picture of the CVTree phylogeny: the Microsporidia is placed in fungi and the Ascomycota and Basidiomycota are resolved as sister taxa that together constitute a clade named Dikarya. Moreover, we also investigated the position of the kingdom by adding gene repertoires of animals, plants and Protozoans. Our results suggested the sister-relationship between fungi and the animal-choanoflagellate clade (data not shown). In the Ascomycota, 3 subphyla are recognized: the Taphrinomycotina is the early-diverging group; the Pezizomycotina includes 4 clades from the "Leotiomyceta" and the Saccharomycotina encompasses the WGD and CTG clades. In the Basidiomycota, monophyly of three subphyla are supported as well.

The novelty of this work can be viewed from the following four aspects: First, the CVTree uses different data from standard methods. Standard methods construct trees using subsets of proteomes or rRNAs as input data. The number is from one to a few hundred. By contrast, the CV method uses all the information of nuclear protein-coding genes. In addition, as we have explained in Introduction, most of the standard multi-gene analyses manually select genes and sites from input data. In contrast, our approach does not need such adjustment, thus circumvent the ambiguity of choosing genes and sites.

Second, the CVTree uses an independent methodology to automatically construct phylogenetic tree. In standard methods, the alignment algorithm, evolutionary model and numerous parameters are needed to be selected and set case-by-case according to the heterogeneity of input data. So far there is no general rule to guide these selections and settings. By contrast, the string-counting strategy of CVTree minimizes arbitrary factors in tree construction. It has only one parameter K ([see Additional file 2] and Methods) and the construction process is automatic. Furthermore, the algorithm is rapid comparing to many standard methods. For instance, it takes only about 1 hour to construct one 82-organism tree on a computer of 2.3 GHz CPU and 4 G RAM for various K values. ([see Additional file 2]).

Third, the CVTree gives novel and stronger supports to the results of standard methods through remarkable consistency between them. We note that such consistency not only confirms the validity of our methods but also strongly support the fungi phylogeny based on the standard methods because such supports come from an independent input dataset and methodology. The supports from CVTree put the current understanding of the fungi phylogeny on a more secure footing. Current kingdom-wide fungi phylogeny has been established on numerous works, each of which investigated a major or minor group and contributed a piece (i.e., local phylogeny) to the whole picture. These works constructed local phylogenies using different input data, site selection strategies, alignment algorithms, evolutionary models and parameters. In other words, the kingdom tree was assembled from local phylogenies that complied with different criteria. However, the feasibility of assembling these pieces together is not self-evident and needs validations. Our CVTree provides such a proper support because all of the relationships therein are inferred under the same criteria. Whenever a group is supported it is reinforced by a "global" picture.

Fourth, novel phylogenetic findings of this work. To the best of our knowledge, the CVTree is so far the only successful kingdom-wide fungi phylogeny constructed by a strategy other than the standard methods. Besides consistency, the current study has shed light on many controversial cases. For example, the CVTree suggests that Dothideomycetes and Eurotiomycetes are sister clades using broader species sampling than previous works [4,10]; that *M. grisea *and the Plectosphaerellaceae group with the Sordariales and Hypocreales, respectively [3,4,10,28]; that the Ajellomycetaceae is a distinct clade from the Onygenaceae [38,39]; and that *A. nidulans *is the earliest diverged among the 8 aspergilli [34,35]. In all of the above examples, contradictions are found between researches done in different periods and it is interesting to note that the CVTree tends to support more recent results. CVTree's support to a certain relationship actually adds weight to it.

The CVTree is robust, to a certain degree, to variations of gene models used in genome annotation: (1) Our experiments showed that randomly adding or dropping ≤ 30% proteins from the whole gene products rarely changed major relationships in the tree ([see Additional file 3]). (2) The gene models of some fungal genomes have been changed rapidly because of lacking of evidences of transcripts and these changes have been reflected in different versions of genome annotation. This enable us to test the topological stability of the CVTree by using different versions of genome annotation to construct trees. We downloaded multiple annotation versions of some organisms, i.e., two versions of *M. grisea *and two of *F. graminearum *and used their combinations to construct trees. Our results showed that all possible combinations generated identical topology ([see Additional file 3] for details). However, the stability was not absolute and differences in gene annotations might alter the tree topology. We found an example (*A. niger*) that different gene annotations of the same species led to two slightly different positions, but it did not affect other species in the tree ([see Additional file 3]).

As a method in development, the current CVTree has some restrictions: (1) The relationship between the branch length of CVTree and that of alignment-based tree is currently unclear. Our simulation experiments have revealed that distance between two CVs is proportional to traditional evolutionary distance when substitutions between two sequences are rare (data not shown). However, because the distance between two CVs is not estimated from site substitutions, the branch length of the CVTree in general does not have simple relationship with the traditional evolutionary distance. As a result we currently can not compare the CVTree with alignment-based tree at the level of scaled branch length. Therefore the current work constructs unscaled tree and only discuss topological relationships. (2) The CVTree may suffer from long-branch attraction and amino acids composition bias. The core of the CV method lies in that it provides an alternative way to construct the distance-matrix. Once the matrix is established, the following tree construction process is performed by NJ method. Many have reported that long-branch attraction and amino acids composition bias may affect the NJ tree topology [49,50]. However, it is not clear how and to what extent the matrix construction process is affected by these errors. (3) Our method is based on whole-genome data, so its sampling scope is restricted by the number of sequenced genomes.

Having been successfully applied to the prokaryotic branches of the TOL, the present research extends the CVTree method to the Eukaryotic kingdom of fungi and provides independent verification of traditional phylogenetic approaches. Further study to cover the whole TOL including all Eukaryotic branches is underway.

## Methods

### The CVTree method

The composition vector method used in this study has been described in previous publications [13-15] so we only give a brief account here. An organism is represented first by a raw composition vector whose components correspond to the number of various overlapping *K*-peptides (for a fixed *K*) in the collection of all protein products encoded in the genome. These 20^*K *^components are put in lexicographic order. In order to highlight the shaping role of selective evolution, the components of a CV are then modified by subtracting a statistical background reflecting the viewpoint of the neutral theory of evolution that mutations happen randomly at molecular level. Our substraction procedure is based on a (*K *- 2)-th order Markov prediction and therefore the minimum *K *starts from 3. The dissimilarity of two species is measured by a correlation distance derived from the corresponding modified CVs. Finally, from the distance matrix thus obtained, a neighbor-joining tree is produced by the PHYLIP package [51]. In the CVTree construction the fixed peptide length *K *controls the resolution of the method. The best choice of K depends on the length of input genomes. Our experiments revealed that *K *= 6 and 5 are the best for prokaryotes and viruses, respectively [13,17]. For fungi, we constructed trees of K = 3 to 10 and evaluated their robustness by 100 bootstrap replicates ([see Additional file 2] and Figure 1). Since the K = 7 topology shows better bootstrap values than others, our discussions in this study are mainly based on it.

### Fungal and outgroup genomes

The collection of protein products from 82 fungal genomes was used in this study (Table 1). We relied on the genome annotations provided by the corresponding sequencing project with *A. niger *being the only exception. This species was annotated in house ([see Additional file 3] and Table 1) by BGF [52,53], an *ab initio *gene prediction tool developed in our laboratory. The last column "Source" in Table 1 indicates the origin of the data: BROAD Institute Fungal Genome Initiative (BROAD-FGI) [54], Department of Energy Joint Genome Institute (JGI) [55], National Center for Biotechnology Information (NCBI) ftp-site [18], Resources for Fungal Comparative Genomics (RFCG) [56] and Fungal Genome Research website (FGR) [57]. A protist choanoflagellate (*Monosiga brevicollis*) and two metazoans (*Caenorhabditis elegans *and *Drosophila melanogaster*) genomes were included as outgroup. The RFCG data used in this study covers all the fungal species which were used in Fitzpatrick *et al *(2006) analysis [4] except for *Candida dubliniensis *because its sequences are not available at RFCG.

**Table 1:** Information of 82 fungi genomes used in this study

Species	Strain	(Sub)Phylum	Source	Download date
*Aspergillus clavatus*	*NRRL1*	Ascomycota	BROAD-FGI	Mar. 2008

*Aspergillus flavus*	*NRRL3357*	Ascomycota	BROAD-FGI	Mar. 2008

*Aspergillus fumigatus*	*Af293*	Ascomycota	BROAD-FGI	Mar. 2008

*Aspergillus nidulans*	*FGSCA4*	Ascomycota	BROAD-FGI	Mar. 2008

*Aspergillus niger*^*a*^	*ATCC1015*	Ascomycota	BROAD-FGI	Mar. 2008

*Aspergillus oryzae*	*RIB40*	Ascomycota	BROAD-FGI	Mar. 2008

*Aspergillus terreus*	*NIH2624*	Ascomycota	BROAD-FGI	Mar. 2008

*Botrytis cinerea*	*B05.10*	Ascomycota	BROAD-FGI	Mar. 2008

*Candida albicans*	*WO-1*	Ascomycota	BROAD-FGI	Mar. 2008

*Candida albicans*	*SC5314*	Ascomycota	BROAD-FGI	Mar. 2008

*Candida glabrata*	*CBS138*	Ascomycota	NCBI	Mar. 2008

*Candida guilliermondii*	*ATCC6260*	Ascomycota	BROAD-FGI	Mar. 2008

*Candida lusitaniae*	*ATCC42720*	Ascomycota	BROAD-FGI	Mar. 2008

*Candida parapsilosis*	*isolate 317*	Ascomycota	BROAD-FGI	Mar. 2008

*Candida tropicalis*	*MYA-3404*	Ascomycota	BROAD-FGI	Mar. 2008

*Chaetomium globosum*	*CBS148.51*	Ascomycota	BROAD-FGI	Mar. 2008

*Coccidioides immitis*	*RS*	Ascomycota	BROAD-FGI	Aug. 2008

*Coccidioides immitis*	*h538.4*	Ascomycota	BROAD-FGI	Aug. 2008

*Coccidioides immitis*	*RMSCC2394*	Ascomycota	BROAD-FGI	Aug. 2008

*Coccidioides immitis*	*RMSCC3703*	Ascomycota	BROAD-FGI	Aug. 2008

*Coccidioides posadasii*	*Silveira*	Ascomycota	BROAD-FGI	Aug. 2008

*Coccidioides posadasii*	*RMSCC3488*	Ascomycota	BROAD-FGI	Aug. 2008

*Cochliobolus heterostrophus*	*C5*	Ascomycota	JGI	Aug. 2008

*Paracoccidioides brasiliensis*	*Pb01*	Ascomycota	BROAD-FGI	Aug. 2008

*Paracoccidioides brasiliensis*	*Pb03*	Ascomycota	BROAD-FGI	Aug. 2008

*Paracoccidioides brasiliensis*	*Pb18*	Ascomycota	BROAD-FGI	Aug. 2008

*Debaryomyces hansenii*	*CBS767*	Ascomycota	BROAD-FGI	Mar. 2008

*Eremothecium gossypii*^*b*^	*ATCC10895*	Ascomycota	NCBI	Mar. 2008

*Fusarium graminearum*	*PH-1*	Ascomycota	BROAD-FGI	Mar. 2008

*Fusarium oxysporum*	*f. sp. lycopersici*	Ascomycota	BROAD-FGI	Mar. 2008

*Fusarium verticillioides*	*7600*	Ascomycota	BROAD-FGI	Mar. 2008

*Histoplasma capsulatum*^*c*^	*WU24(NAm1)*	Ascomycota	BROAD-FGI	Mar. 2008

*Kluyveromyces lactis*	*NRRLY-1140*	Ascomycota	BROAD-FGI	Mar. 2008

*Kluyveromyces waltii*	*NCYC 2644*	Ascomycota	RFCG	Mar. 2008

*Lodderomyces elongisporus*	*NRRLYB-4239*	Ascomycota	BROAD-FGI	Mar. 2008

*Magnaporthe grisea*	*70–15*	Ascomycota	BROAD-FGI	Mar. 2008

*Mycosphaerella fijiensis*	*CIRAD86*	Ascomycota	JGI	Mar. 2008

*Mycosphaerella graminicola*	*IPO323*	Ascomycota	JGI	Mar. 2008

*Nectria haematococca*^*d*^	*MPVI*	Ascomycota	JGI	Mar. 2008

*Neosartorya fischeri*	*NRRL181*	Ascomycota	BROAD-FGI	Mar. 2008

*Neurospora crassa*	*OR74A*	Ascomycota	BROAD-FG	Mar. 2008I

*Pyrenophora tritici-repentis*	*Pt-1C-BFP*	Ascomycota	BROAD-FGI	Aug. 2008

*Pichia stipitis*	*CBS6054*	Ascomycota	JGI	Mar. 2008

*Podospora anserina*	*DSM980*	Ascomycota	RFCG	Mar. 2008

*Saccharomyces cerevisiae*	*S288C*	Ascomycota	NCBI	Mar. 2008

*Saccharomyces cerevisiae*	*rm11-1a*	Ascomycota	BROAD-FGI	Mar. 2008

*Saccharomyces cerevisiae*	*YJM789*	Ascomycota	RFCG	Mar. 2008

*Saccharomyces paradoxus*	*NRRLY-17217*	Ascomycota	RFCG	Mar. 2008

*Saccharomyces mikatae*	*IFO1815*	Ascomycota	RFCG	Mar. 2008

*Saccharomyces kudriavzevii*	*IFO1802*	Ascomycota	RFCG	Mar. 2008

*Saccharomyces bayanus*	*MCYC623*	Ascomycota	RFCG	Mar. 2008

*Saccharomyces castellii*	*NRRLY-12630*	Ascomycota	RFCG	Mar. 2008

*Saccharomyces kluyveri*	*NRRL Y-12651*	Ascomycota	RFCG	Mar. 2008

*Schizosaccharomyces japonicus*	*yFS275*	Ascomycota	BROAD-FGI	Mar. 2008

*Schizosaccharomyces octosporus*	*yFS286*	Ascomycota	FGR	Aug. 2008

*Schizosaccharomyces pombe*	*972h-*	Ascomycota	BROAD-FGI	Mar. 2008

*Sclerotinia sclerotiorum*	*1980*	Ascomycota	BROAD-FGI	Mar. 2008

*Stagonospora nodorum*	*SN15*	Ascomycota	BROAD-FGI	Mar. 2008

*Trichoderma atroviride*	*IMI202040*	Ascomycota	JGI	Aug. 2008

*Trichoderma reesei*	*QM6a*	Ascomycota	JGI	Mar. 2008

*Trichoderma virens*	*Gv29-8*	Ascomycota	JGI	Mar. 2008

*Uncinocarpus reesii*	*1704*	Ascomycota	BROAD-FGI	Mar. 2008

*Verticillium dahliae*	*VdLs.17*	Ascomycota	BROAD-FGI	Aug. 2008

*Verticillium albo-atrum*	*VaMs.102*	Ascomycota	BROAD-FGI	Aug. 2008

*Yarrowia lipolytica*	*CLIB122*	Ascomycota	NCBI	Mar. 2008


*Coprinus cinereus*	*Okayama7#130*	Basidiomycota	BROAD-FGI	Mar. 2008

*Cryptococcus neoformans*	*serotypeA, strainH99*	Basidiomycota	BROAD-FGI	Mar. 2008

*Cryptococcus neoformans*	*serotypeD, strainJEC21*	Basidiomycota	NCBI	Mar. 2008

*Cryptococcus gattii*	*serotypeB, strainWM276*	Basidiomycota	RFCG	Mar. 2008

*Cryptococcus gattii*	*serotypeB/C, strainR265*	Basidiomycota	RFCG	Mar. 2008

*Laccaria bicolor*	*S238N-H82*	Basidiomycota	JGI	Mar. 2008

*Malassezia globosa*	*CBS7966*	Basidiomycota	FGR	Aug. 2008

*Phanerochaete chrysosporium*	*RP-78*	Basidiomycota	JGI	Mar. 2008

*Postia placenta*		Basidiomycota	JGI	Mar. 2008

*Puccinia graminis*	*f. sp. tritici*	Basidiomycota	BROAD-FGI	Mar. 2008

*Sporobolomyces roseus*		Basidiomycota	JGI	Mar. 2008

*Ustilago maydis*	*521*	Basidiomycota	BROAD-FGI	Mar. 2008

*Batrachochytrium dendrobatidis*	*JAM81*	Chytridiomycota	JGI	Apr. 2008

*Batrachochytrium dendrobatidis*	*JEL423*	Chytridiomycota	BROAD-FGI	Mar. 2008


*Rhizopus oryzae*	*RA99–880*	Mucoromycotina	BROAD-FGI	Mar. 2008

*Phycomyces blakesleeanus*		Mucoromycotina	JGI	Mar. 2008


*Encephalitozoon cuniculi*	*GB-M1*	Microsporidia	NCBI	Mar. 2008

^*a *^annotated in house ([See Additional file 3])

^*d *^synonym: *Ashbya gossypii*

^*c *^teleomorph: *Ajellomyces capsulata*

^*d *^anamorph: *Fusarium solani*

### Fungal phylogeny references

In order to integrate the multi-laboratory efforts in fungal molecular phylogeny and taxonomy, an Assembling the Fungal Tree of Life (AFTOL) project was launched in 2002 [58]. As a preliminary but successful outcome of AFTOL, Hibbett *et al*. have published a higher-level fungal phylogenetic classification [5]. It is a classification as more than two lower taxa belonging to a higher taxon may be juxtaposed in this scheme and seven Linnaeus ranks (kingdom, subkingdom, phylum, subphylum, class, subclass, and order) are used. It is phylogenetic as well because emphasis has been put on each included taxon being a monophyletic group.

This classification allows us to use terms of taxonomy when analyzing phylogenetic relationships. However, it is worth noting that our work concerns phylogeny, but not taxonomy. Accordingly, taxonomic terms such as "higher-level" or "class-level" clades, "three subphyla" and "the order Sordariales" should be understood phylogenetically. In other words, taxa in this study are not used as labels of hierarchical taxonomic ranks but only as names of clades. Higher or lower levels actually mean major or minor clades. A clade being called a class or an order merely means that it forms a monophyletic branch and has been named in that way in some previous publications.

The present study infers phylogenetic relationships from whole-genome data of fungi without using sequence alignment. We use the Hibbett *et al *paper [5] as a major reference to phylogenetic classification at higher-than-order levels. When it comes to compare the branching pattern of originally juxtaposed taxa we refer to recent phylogenetic studies that utilize the standard methods.

Regarding phylogeny at lower-than-order levels, we used the following three publicly accessible databases as references. Index Fungorum [27] provides an up-to-date phylogenetic classification of fungi. Focusing on the Ascomycota, Myconet [59] regularly updates its classification. Another popular taxonomy database is NCBI taxonomy browser [60], which integrates classifications from various sources and gives useful information as well. If at a certain node the resolution is not high enough or when discrepancy between the CVTree phylogeny and current classification was found, we refer to recent publications for more information.

## Abbreviations

CV: the composition vector; CVTree: the phylogenetic tree obtained by using CV method; TOL: Tree of Life; WGD: whole-genome duplication; AFTOL: the Assembling the Fungal Tree of Life Project; LSU: large subunit; rDNA: ribosomal RNA-encoding DNA.

## Authors' contributions

BH and HW designed the study, carried out the molecular phylogenetic analysis, and drafted the manuscript. HW and LG collected the genome dataset. ZX and LG developed the CVTree program. All authors read and approved the final manuscript.

## Supplementary Material

Additional file 1 **Previous phylogeny of Aspergilli and Hypocreales species**. This file contains two diagrams showing (1) the relationships among 8 aspergilli according to [36] and (2) NCBI classification of the 7 Hypocreales species [18]. Click here for file

Additional file 2 **The Selection of peptide length K**. This file contains to parts: (1) runtime of the CVTree program with different K values; (2) tree with K values varied from 3 to 10. The results suggest that the tree with K = 7 is the best one. Click here for file

Additional file 3 **Statistical testing of the CVTree**. This file contains two parts: (1) the bootstrap of CVTree; (2) The robustness of the CVTree to different versions of genome annotation. Click here for file
