# Supplementary material for: A fungal phylogeny based on 82 complete genomes using the composition vector method
Source: BMC Evol Biol. 2009 Aug 10;9:195. doi: 10.1186/1471-2148-9-195 (PMC3087519; doi:10.1186/1471-2148-9-195)
Supplement: Additional file 1 — Previous phylogeny of Aspergilli and Hypocreales species. This file contains two diagrams showing (1) the relationships among 8 aspergilli according to [36] and (2) NCBI classification of the 7 Hypocreales species [18]. [file 1471-2148-9-195-S1.pdf]

Additional file 1 to:
“A fungal phylogeny based on 82 complete genomes using the composition vector method”

Hao Wang, Zhao Xu, Lei Gao and Bailin Hao

Figure S1 - Previous results of Aspergilli and Hypocreales species

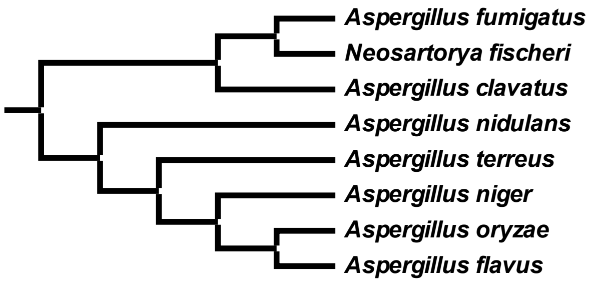

(a) Phylogeny of 8 Aspergilli according to Peterson (2000) study.

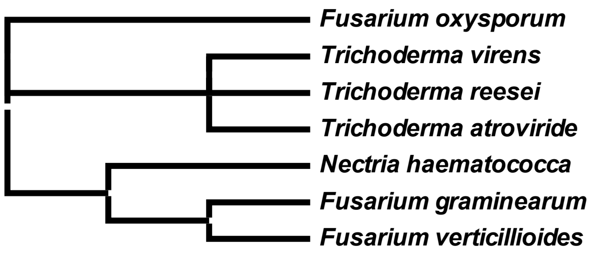

(b) Classification of 7 sequenced Hypocreales species according to NCBI Taxonomy browser.

Figure S1: Previous results of the relationships of Aspergilli and Hypocreales species
