# Supplementary material for: A fungal phylogeny based on 82 complete genomes using the composition vector method
Source: BMC Evol Biol. 2009 Aug 10;9:195. doi: 10.1186/1471-2148-9-195 (PMC3087519; doi:10.1186/1471-2148-9-195)
Supplement: Additional file 2 — The Selection of peptide length K. This file contains to parts: (1) runtime of the CVTree program with different K values; (2) tree with K values varied from 3 to 10. The results suggest that the tree with K = 7 is the best one. [file 1471-2148-9-195-S2.pdf]

Additional file 2 to:
"A fungal phylogeny based on 82 complete genomes using the composition vector method"

Hao Wang, Zhao Xu, Lei Gao and Bailin Hao

Runtime of the CVTree

We have published a webserver for the CVTree algorithm into which the 82 fungi proteomes were integrated (`http://tlife.fudan.edu.cn/cvtree/`). We have run the program with K ranging from 3 to 10 and analyzed the performance. According to Table S1, The runtime varies with K, reaching the maximum when K=6. That runtime depends on the number of non-zero components of CV can be explained by the fact that the most time-consuming part in our current program is the harddisk I/O process reading and writing these components.

Table S1: Runtime of the CVTree. [a] the number of components of CV. [b] the mean number of non-zero components of CV. [c] the experiments were implemented on a computer of 2.3 GHz CPU and 4 G RAM

K	3	4	5	6	7	8	9	10
N^a ($\times 10^6$)	0.008	0.16	3.2	64	1280	25600	512000	10240000
M^b ($\times 10^6$)	0.008	0.16	3.0	19.9	12.3	4.77	3.95	3.88
Runtime^c (min)	0.9	2.9	23.3	65.9	56.3	60.9	50.7	56.8

The best tree is obtained with K=7

The parameter K is the "resolution" controller of evolutionary information. Too small or too large K values fail to extract proper evolutionary features representing the organisms. CVTrees of K = 3 to 6 and 8 to 10 are shown in Figure S2.

By comparing these trees to the K=7 topology (Figure 1 in the main text), we found that the bootstrap values (BPs) in trees improved with K increasing from 3 to 7 but deteriorated when K continued to increase. That is, the K=7 topology has better bootstrap values than all other trees investigated.

According to Figure S2 and Figure 1, it is obvious that both the topology and BPs become better with the K value increasing from 3 to 5 and worse when K longer than 7. Although K=5, 6 and 7 all produce reasonable topology and BPs at a glance, one observes the same improving process with the K increasing from 5 to 7 when conducting

careful comparison (see Figure S2 and Figure 1): (1) At the level of Phylum, the K=5, 6, 7 trees all provides well-organized structure. The K=5 tree supports the Dikarya with a BP=93%, while the K=6 and 7 trees with BP=100%. (2) The K=5 and 6 trees give a sister relationship of the subphylum Taphrinomycotina and Saccharomycotina to the exclusion of the Pezizomycotina (see figure S2). This conflicts to, as we have mentioned in the main text, many resent researches that put the fission yeasts as the basal lineage of the Ascomycota. By contrast, the K=7 tree recovers the (Taphrinomycotina, (Saccharomycotina, Pezizomycotina)) structure with high bootstrap value (100% and 99%). (3) In the Basidiomycota, the subphylum-level relationships within the Basidiomycota in the K=5 tree is far less reasonable because organisms from the three subphyla mix together. The K=6 tree recovers the Agaricomycotina but fails to represent the monophyly of the well-supported subphyla Pucciniomycotina and Ustilaginomycotina by misplacing *Sporobolomyces roseus*. In contrast, the K=7 tree well supports (BP=100% and 99%) the three subphyla. (4) In the Saccharomycotina, the placement of *S. kudriavzevii* is modified and gains higher supports with K increasing from 5 to 7. (5) In the *Aspergillus*, the placement of *A. niger* and *A. terreus* is modified and gains higher supports with K increasing from 5 to 7.

From the trees showed in Figure S2 and Figure 1, one can easily see a trend that both bootstrap values and the quality of tree topology become better and better when K increases from 3 to 7 but worse and worse when K increases from 7 to 10. Here we must explain what we mean by "the quality of tree topology": in the traditional SSU rRNA or few-gene based whole-genome phylogeny, the quality of a tree is judged mostly by stability and self-consistent arguments (by statistical resampling, e.g., bootstrapping or Jack-knifing). However, we take the century-long taxonomy, especially the recent results based on SSU rRNA analysis as EXPERIMENTAL facts and view the CVTree as THEORETICAL construction. The quality of a tree is judged by how close THEORY agrees with EXPERIMENT. We choose K=7 not only based on its best BP values but also on its best quality (in the above sense).

K=3

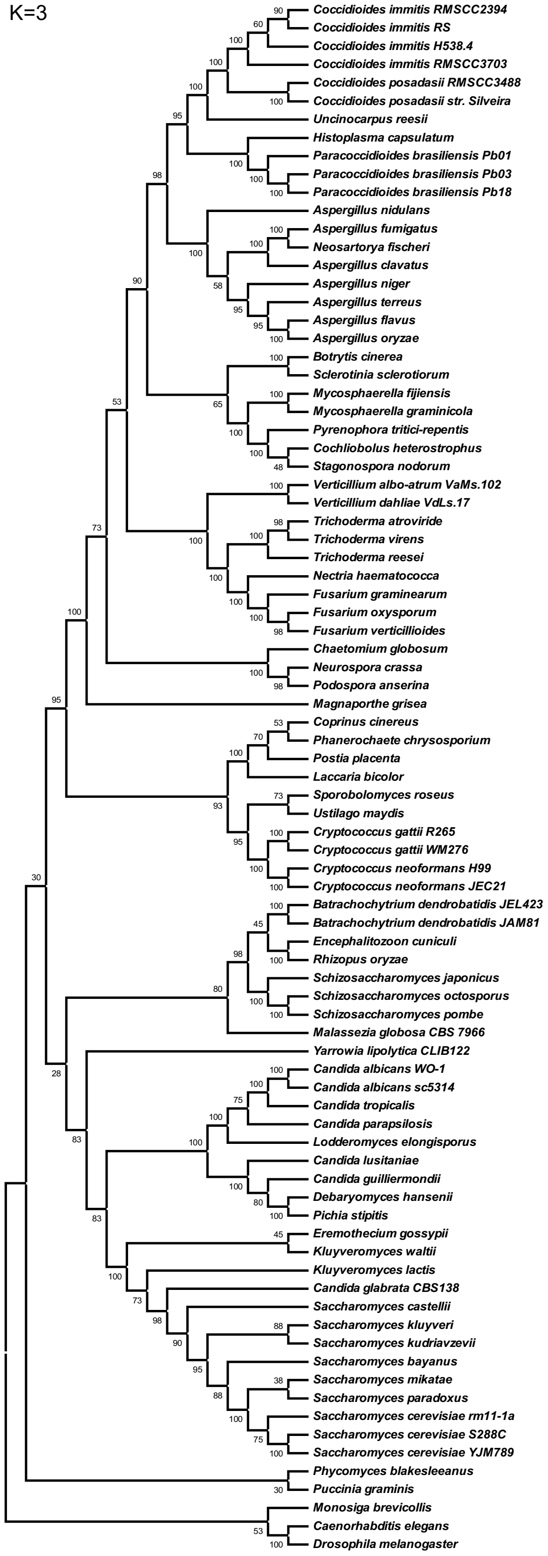
Coccidioides immitis RMSCC2394
Coccidioides immitis RS
Coccidioides immitis H538.4
Coccidioides immitis RMSCC3703
Coccidioides posadasii RMSCC3488
Coccidioides posadasii str. Silveira
Uncinocarpus reesii
Histoplasma capsulatum
Paracoccidioides brasiliensis Pb01
Paracoccidioides brasiliensis Pb03
Paracoccidioides brasiliensis Pb18
Aspergillus nidulans
Aspergillus fumigatus
Neosartorya fischeri
Aspergillus clavatus
Aspergillus niger
Aspergillus terreus
Aspergillus flavus
Aspergillus oryzae
Botrytis cinerea
Sclerotinia sclerotiorum
Mycosphaerella fijiensis
Mycosphaerella graminicola
Pyrenophora tritici-repentis
Cochliobolus heterostrophus
Stagonospora nodorum
Verticillium albo-atrum VaMs.102
Verticillium dahliae VdLs.17
Trichoderma atroviride
Trichoderma virens
Trichoderma reesei
Nectria haematococca
Fusarium graminearum
Fusarium oxysporum
Fusarium verticillioides
Chaetomium globosum
Neurospora crassa
Podospora anserina
Magnaporthe grisea
Coprinus cinereus
Phanerochaete chrysosporium
Postia placenta
Laccaria bicolor
Sporobolomyces roseus
Ustilago maydis
Cryptococcus gattii R265
Cryptococcus gattii WM276
Cryptococcus neoformans H99
Cryptococcus neoformans JEC21
Batrachochytrium dendrobatidis JEL423
Batrachochytrium dendrobatidis JAM81
Encephalitozoon cuniculi
Rhizopus oryzae
Schizosaccharomyces japonicus
Schizosaccharomyces octosporus
Schizosaccharomyces pombe
Malassezia globosa CBS 7966
Yarrowia lipolytica CLIB122
Candida albicans WO-1
Candida albicans sc5314
Candida tropicalis
Candida parapsilosis
Lodderomyces elongisporus
Candida lusitaniae
Candida guilliermondii
Debaryomyces hansenii
Pichia stipitis
Eremothecium gossypii
Kluyveromyces waltii
Kluyveromyces lactis
Candida glabrata CBS138
Saccharomyces castellii
Saccharomyces kluyveri
Saccharomyces kudriavzevii
Saccharomyces bayanus
Saccharomyces mikatae
Saccharomyces paradoxus
Saccharomyces cerevisiae rm11-1a
Saccharomyces cerevisiae S288C
Saccharomyces cerevisiae YJM789
Phycomyces blakesleeanus
Puccinia graminis
Monosiga brevicollis
Caenorhabditis elegans
Drosophila melanogaster

K=4

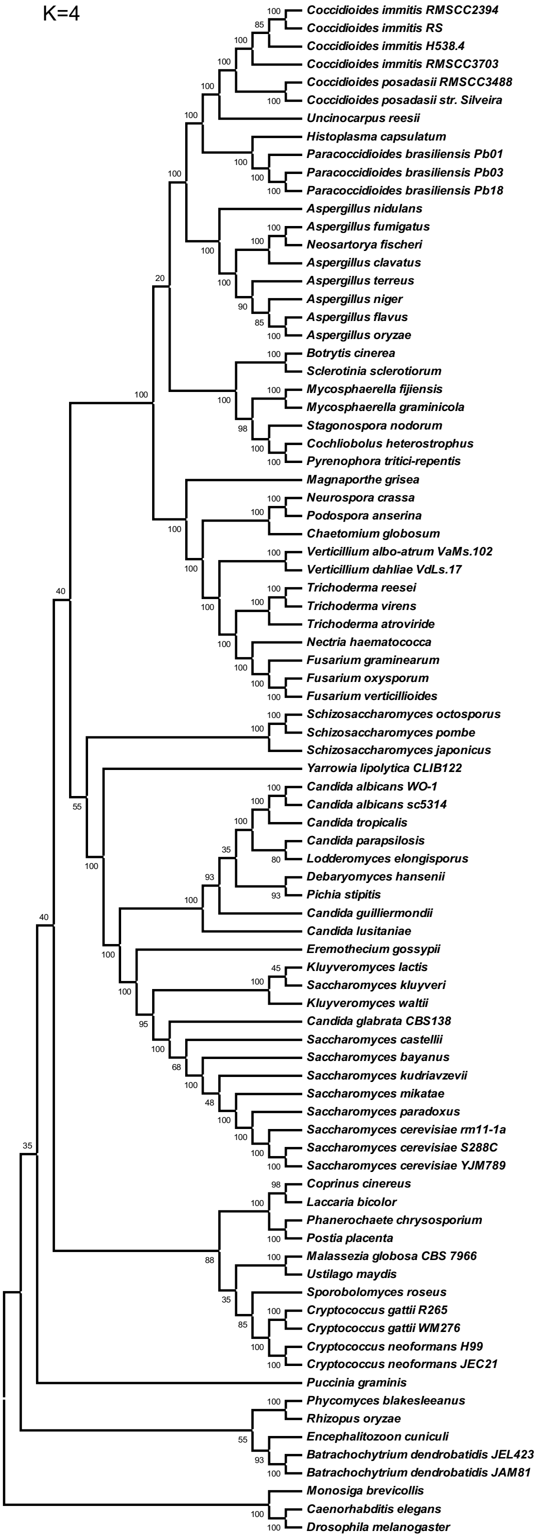
Coccidioides immitis RMSCC2394
Coccidioides immitis RS
Coccidioides immitis H538.4
Coccidioides immitis RMSCC3703
Coccidioides posadasii RMSCC3488
Coccidioides posadasii str. Silveira
Uncinocarpus reesii
Histoplasma capsulatum
Paracoccidioides brasiliensis Pb01
Paracoccidioides brasiliensis Pb03
Paracoccidioides brasiliensis Pb18
Aspergillus nidulans
Aspergillus fumigatus
Neosartorya fischeri
Aspergillus clavatus
Aspergillus terreus
Aspergillus niger
Aspergillus flavus
Aspergillus oryzae
Botrytis cinerea
Sclerotinia sclerotiorum
Mycosphaerella fijiensis
Mycosphaerella graminicola
Stagonospora nodorum
Cochliobolus heterostrophus
Pyrenophora tritici-repentis
Magnaporthe grisea
Neurospora crassa
Podospora anserina
Chaetomium globosum
Verticillium albo-atrum VaMs.102
Verticillium dahliae VdLs.17
Trichoderma reesei
Trichoderma virens
Trichoderma atroviride
Nectria haematococca
Fusarium graminearum
Fusarium oxysporum
Fusarium verticillioides
Schizosaccharomyces octosporus
Schizosaccharomyces pombe
Schizosaccharomyces japonicus
Yarrowia lipolytica CLIB122
Candida albicans WO-1
Candida albicans sc5314
Candida tropicalis
Candida parapsilosis
Lodderomyces elongisporus
Debaryomyces hansenii
Pichia stipitis
Candida guilliermondii
Candida lusitaniae
Eremothecium gossypii
Kluyveromyces lactis
Saccharomyces kluyveri
Kluyveromyces waltii
Candida glabrata CBS138
Saccharomyces castellii
Saccharomyces bayanus
Saccharomyces kudriavzevii
Saccharomyces mikatae
Saccharomyces paradoxus
Saccharomyces cerevisiae rm11-1a
Saccharomyces cerevisiae S288C
Saccharomyces cerevisiae YJM789
Coprinus cinereus
Laccaria bicolor
Phanerochaete chrysosporium
Postia placenta
Malassezia globosa CBS 7966
Ustilago maydis
Sporobolomyces roseus
Cryptococcus gattii R265
Cryptococcus gattii WM276
Cryptococcus neoformans H99
Cryptococcus neoformans JEC21
Puccinia graminis
Phycomyces blakesleeanus
Rhizopus oryzae
Encephalitozoon cuniculi
Batrachochytrium dendrobatidis JEL423
Batrachochytrium dendrobatidis JAM81
Monosiga brevicollis
Caenorhabditis elegans
Drosophila melanogaster

K=5

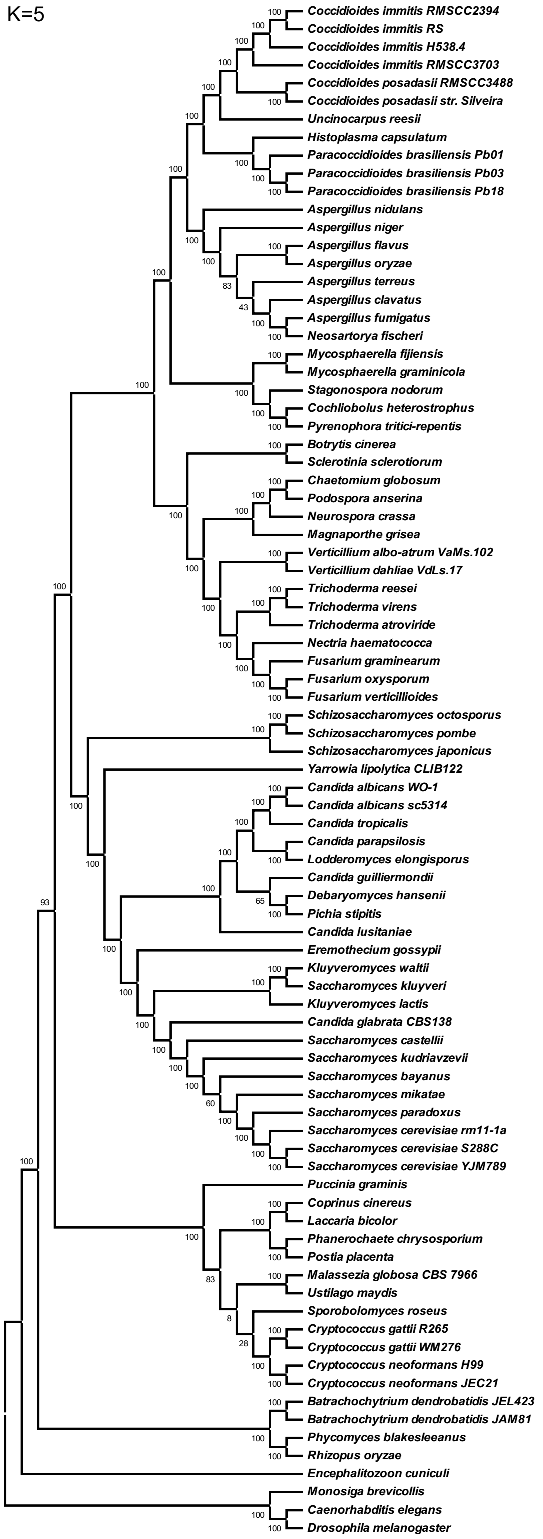
Coccidioides immitis RMSCC2394
Coccidioides immitis RS
Coccidioides immitis H538.4
Coccidioides immitis RMSCC3703
Coccidioides posadasii RMSCC3488
Coccidioides posadasii str. Silveira
Uncinocarpus reesii
Histoplasma capsulatum
Paracoccidioides brasiliensis Pb01
Paracoccidioides brasiliensis Pb03
Paracoccidioides brasiliensis Pb18
Aspergillus nidulans
Aspergillus niger
Aspergillus flavus
Aspergillus oryzae
Aspergillus terreus
Aspergillus clavatus
Aspergillus fumigatus
Neosartorya fischeri
Mycosphaerella fijiensis
Mycosphaerella graminicola
Stagonospora nodorum
Cochliobolus heterostrophus
Pyrenophora tritici-repentis
Botrytis cinerea
Sclerotinia sclerotiorum
Chaetomium globosum
Podospora anserina
Neurospora crassa
Magnaporthe grisea
Verticillium albo-atrum VaMs.102
Verticillium dahliae VdLs.17
Trichoderma reesei
Trichoderma virens
Trichoderma atroviride
Nectria haematococca
Fusarium graminearum
Fusarium oxysporum
Fusarium verticillioides
Schizosaccharomyces octosporus
Schizosaccharomyces pombe
Schizosaccharomyces japonicus
Yarrowia lipolytica CLIB122
Candida albicans WO-1
Candida albicans sc5314
Candida tropicalis
Candida parapsilosis
Lodderomyces elongisporus
Candida guilliermondii
Debaryomyces hansenii
Pichia stipitis
Candida lusitaniae
Eremothecium gossypii
Kluyveromyces waltii
Saccharomyces kluyveri
Kluyveromyces lactis
Candida glabrata CBS138
Saccharomyces castellii
Saccharomyces kudriavzevii
Saccharomyces bayanus
Saccharomyces mikatae
Saccharomyces paradoxus
Saccharomyces cerevisiae rm11-1a
Saccharomyces cerevisiae S288C
Saccharomyces cerevisiae YJM789
Puccinia graminis
Coprinus cinereus
Laccaria bicolor
Phanerochaete chrysosporium
Postia placenta
Malassezia globosa CBS 7966
Ustilago maydis
Sporobolomyces roseus
Cryptococcus gattii R265
Cryptococcus gattii WM276
Cryptococcus neoformans H99
Cryptococcus neoformans JEC21
Batrachochytrium dendrobatidis JEL423
Batrachochytrium dendrobatidis JAM81
Phycomyces blakesleeanus
Rhizopus oryzae
Encephalitozoon cuniculi
Monosiga brevicollis
Caenorhabditis elegans
Drosophila melanogaster

K=6

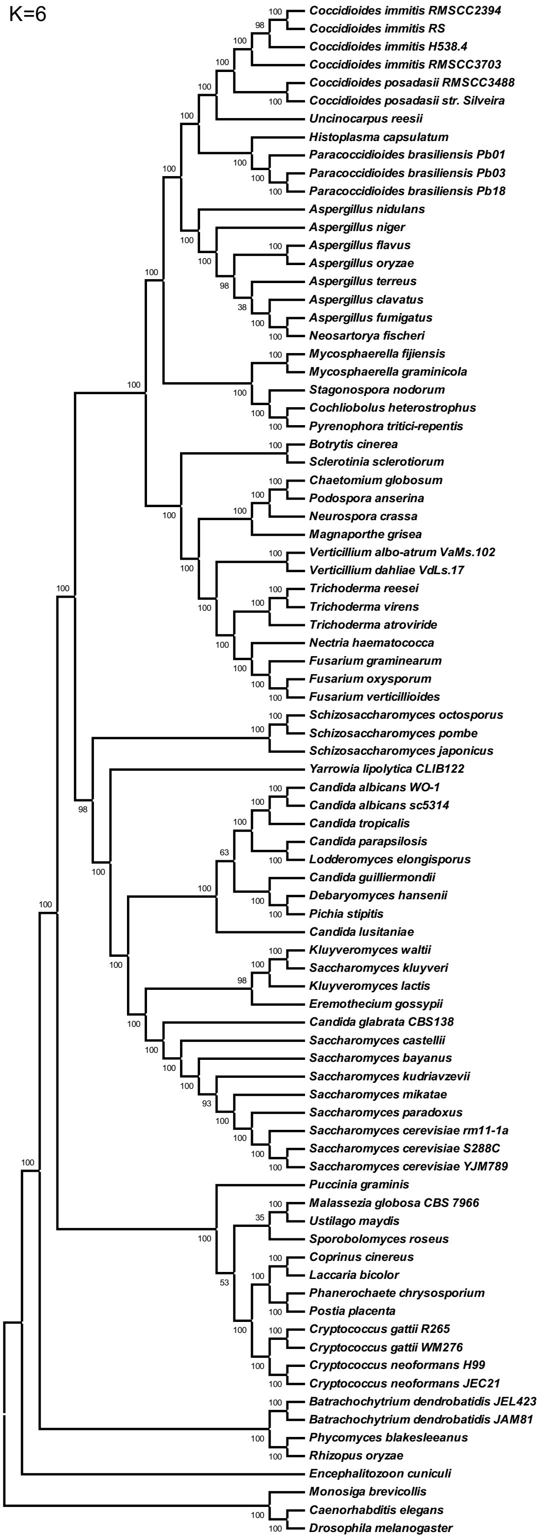
Coccidioides immitis RMSCC2394
Coccidioides immitis RS
Coccidioides immitis H538.4
Coccidioides immitis RMSCC3703
Coccidioides posadasii RMSCC3488
Coccidioides posadasii str. Silveira
Uncinocarpus reesii
Histoplasma capsulatum
Paracoccidioides brasiliensis Pb01
Paracoccidioides brasiliensis Pb03
Paracoccidioides brasiliensis Pb18
Aspergillus nidulans
Aspergillus niger
Aspergillus flavus
Aspergillus oryzae
Aspergillus terreus
Aspergillus clavatus
Aspergillus fumigatus
Neosartorya fischeri
Mycosphaerella fijiensis
Mycosphaerella graminicola
Stagonospora nodorum
Cochliobolus heterostrophus
Pyrenophora tritici-repentis
Botrytis cinerea
Sclerotinia sclerotiorum
Chaetomium globosum
Podospora anserina
Neurospora crassa
Magnaporthe grisea
Verticillium albo-atrum VaMs.102
Verticillium dahliae VdLs.17
Trichoderma reesei
Trichoderma virens
Trichoderma atroviride
Nectria haematococca
Fusarium graminearum
Fusarium oxysporum
Fusarium verticillioides
Schizosaccharomyces octosporus
Schizosaccharomyces pombe
Schizosaccharomyces japonicus
Yarrowia lipolytica CLIB122
Candida albicans WO-1
Candida albicans sc5314
Candida tropicalis
Candida parapsilosis
Lodderomyces elongisporus
Candida guilliermondii
Debaryomyces hansenii
Pichia stipitis
Candida lusitaniae
Kluyveromyces waltii
Saccharomyces kluyveri
Kluyveromyces lactis
Eremothecium gossypii
Candida glabrata CBS138
Saccharomyces castellii
Saccharomyces bayanus
Saccharomyces kudriavzevii
Saccharomyces mikatae
Saccharomyces paradoxus
Saccharomyces cerevisiae rm11-1a
Saccharomyces cerevisiae S288C
Saccharomyces cerevisiae YJM789
Puccinia graminis
Malassezia globosa CBS 7966
Ustilago maydis
Sporobolomyces roseus
Coprinus cinereus
Laccaria bicolor
Phanerochaete chrysosporium
Postia placenta
Cryptococcus gattii R265
Cryptococcus gattii WM276
Cryptococcus neoformans H99
Cryptococcus neoformans JEC21
Batrachochytrium dendrobatidis JEL423
Batrachochytrium dendrobatidis JAM81
Phycomyces blakesleeanus
Rhizopus oryzae
Encephalitozoon cuniculi
Monosiga brevicollis
Caenorhabditis elegans
Drosophila melanogaster

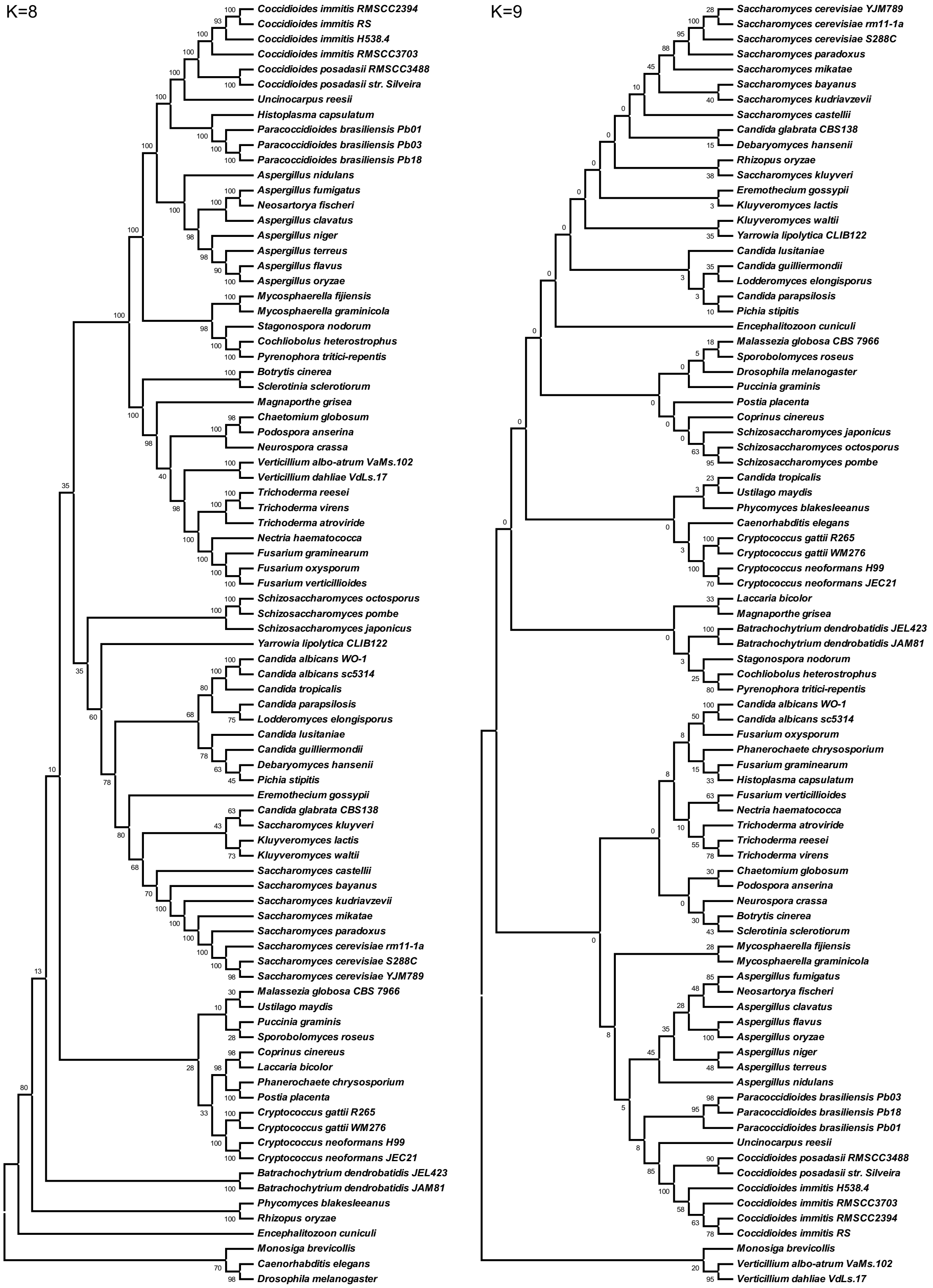
K=8
Coccidioides immitis RMSCC2394
Coccidioides immitis RS
Coccidioides immitis H538.4
Coccidioides immitis RMSCC3703
Coccidioides posadasii RMSCC3488
Coccidioides posadasii str. Silveira
Uncinocarpus reesii
Histoplasma capsulatum
Paracoccidioides brasiliensis Pb01
Paracoccidioides brasiliensis Pb03
Paracoccidioides brasiliensis Pb18
Aspergillus nidulans
Aspergillus fumigatus
Neosartorya fischeri
Aspergillus clavatus
Aspergillus niger
Aspergillus terreus
Aspergillus flavus
Aspergillus oryzae
Mycosphaerella fijiensis
Mycosphaerella graminicola
Stagonospora nodorum
Cochliobolus heterostrophus
Pyrenophora tritici-repentis
Botrytis cinerea
Sclerotinia sclerotiorum
Magnaporthe grisea
Chaetomium globosum
Podospora anserina
Neurospora crassa
Verticillium albo-atrum VaMs.102
Verticillium dahliae VdLs.17
Trichoderma reesei
Trichoderma virens
Trichoderma atroviride
Nectria haematococca
Fusarium graminearum
Fusarium oxysporum
Fusarium verticillioides
Schizosaccharomyces octosporus
Schizosaccharomyces pombe
Schizosaccharomyces japonicus
Yarrowia lipolytica CLIB122
Candida albicans WO-1
Candida albicans sc5314
Candida tropicalis
Candida parapsilosis
Lodderomyces elongisporus
Candida lusitaniae
Candida guilliermondii
Debaryomyces hansenii
Pichia stipitis
Eremothecium gossypii
Candida glabrata CBS138
Saccharomyces kluyveri
Kluyveromyces lactis
Kluyveromyces waltii
Saccharomyces castellii
Saccharomyces bayanus
Saccharomyces kudriavzevii
Saccharomyces mikatae
Saccharomyces paradoxus
Saccharomyces cerevisiae rm11-1a
Saccharomyces cerevisiae S288C
Saccharomyces cerevisiae YJM789
Malassezia globosa CBS 7966
Ustilago maydis
Puccinia graminis
Sporobolomyces roseus
Coprinus cinereus
Laccaria bicolor
Phanerochaete chrysosporium
Postia placenta
Cryptococcus gattii R265
Cryptococcus gattii WM276
Cryptococcus neoformans H99
Cryptococcus neoformans JEC21
Batrachochytrium dendrobatidis JEL423
Batrachochytrium dendrobatidis JAM81
Phycomyces blakesleeanus
Rhizopus oryzae
Encephalitozoon cuniculi
Monosiga brevicollis
Caenorhabditis elegans
Drosophila melanogaster
K=9
Saccharomyces cerevisiae YJM789
Saccharomyces cerevisiae rm11-1a
Saccharomyces cerevisiae S288C
Saccharomyces paradoxus
Saccharomyces mikatae
Saccharomyces bayanus
Saccharomyces kudriavzevii
Saccharomyces castellii
Candida glabrata CBS138
Debaryomyces hansenii
Rhizopus oryzae
Saccharomyces kluyveri
Eremothecium gossypii
Kluyveromyces lactis
Kluyveromyces waltii
Yarrowia lipolytica CLIB122
Candida lusitaniae
Candida guilliermondii
Lodderomyces elongisporus
Candida parapsilosis
Pichia stipitis
Encephalitozoon cuniculi
Malassezia globosa CBS 7966
Sporobolomyces roseus
Drosophila melanogaster
Puccinia graminis
Postia placenta
Coprinus cinereus
Schizosaccharomyces japonicus
Schizosaccharomyces octosporus
Schizosaccharomyces pombe
Candida tropicalis
Ustilago maydis
Phycomyces blakesleeanus
Caenorhabditis elegans
Cryptococcus gattii R265
Cryptococcus gattii WM276
Cryptococcus neoformans H99
Cryptococcus neoformans JEC21
Laccaria bicolor
Magnaporthe grisea
Batrachochytrium dendrobatidis JEL423
Batrachochytrium dendrobatidis JAM81
Stagonospora nodorum
Cochliobolus heterostrophus
Pyrenophora tritici-repentis
Candida albicans WO-1
Candida albicans sc5314
Fusarium oxysporum
Phanerochaete chrysosporium
Fusarium graminearum
Histoplasma capsulatum
Fusarium verticillioides
Nectria haematococca
Trichoderma atroviride
Trichoderma reesei
Trichoderma virens
Chaetomium globosum
Podospora anserina
Neurospora crassa
Botrytis cinerea
Sclerotinia sclerotiorum
Mycosphaerella fijiensis
Mycosphaerella graminicola
Aspergillus fumigatus
Neosartorya fischeri
Aspergillus clavatus
Aspergillus flavus
Aspergillus oryzae
Aspergillus niger
Aspergillus terreus
Aspergillus nidulans
Paracoccidioides brasiliensis Pb03
Paracoccidioides brasiliensis Pb18
Paracoccidioides brasiliensis Pb01
Uncinocarpus reesii
Coccidioides posadasii RMSCC3488
Coccidioides posadasii str. Silveira
Coccidioides immitis H538.4
Coccidioides immitis RMSCC3703
Coccidioides immitis RMSCC2394
Coccidioides immitis RS
Monosiga brevicollis
Verticillium albo-atrum VaMs.102
Verticillium dahliae VdLs.17

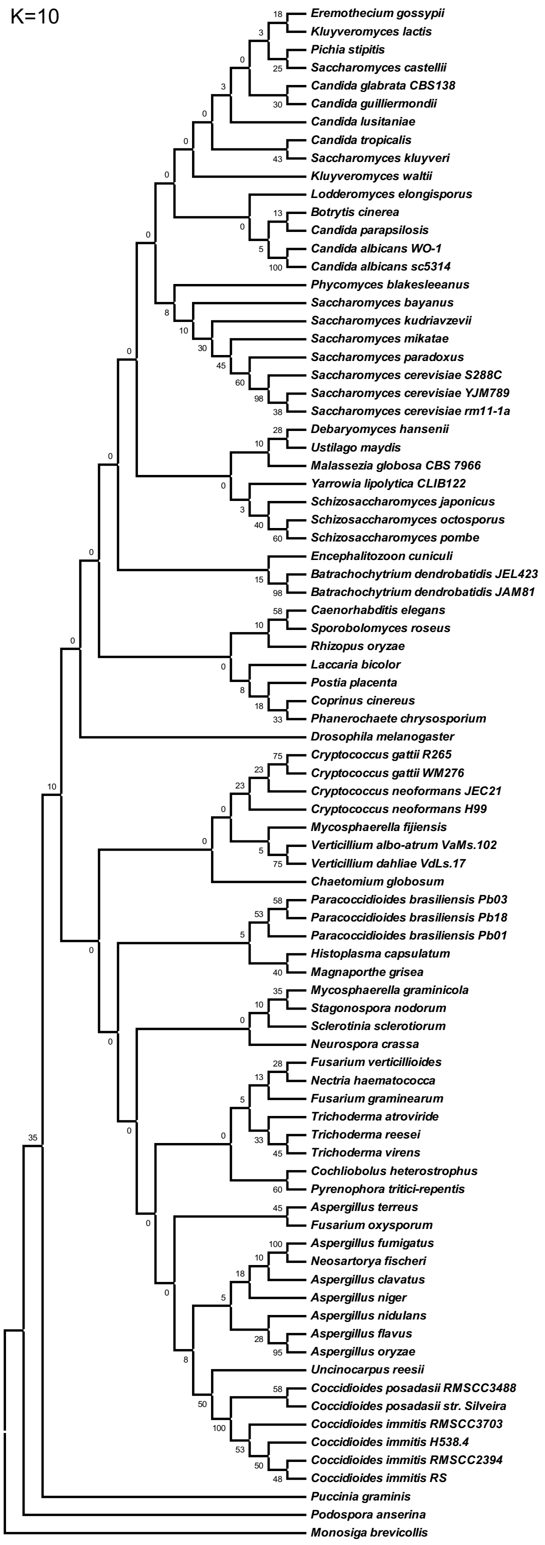

Figure S2: CVTrees with K=3 to 6 and 8 to 10. In each tree, BPs (100 bootstrap replicates) are reported as percentages.
