# Supplementary material for: A fungal phylogeny based on 82 complete genomes using the composition vector method
Source: BMC Evol Biol. 2009 Aug 10;9:195. doi: 10.1186/1471-2148-9-195 (PMC3087519; doi:10.1186/1471-2148-9-195)
Supplement: Additional file 3 — Statistical testing of the CVTree. This file contains two parts: (1) the bootstrap of CVTree; (2) The robustness of the CVTree to different versions of genome annotation. [file 1471-2148-9-195-S3.pdf]

Additional file 3 to: "A fungal phylogeny based on 82 complete genomes using the composition vector method"

Hao Wang, Zhao Xu, Lei Gao and Bailin Hao

The bootstrap of the CVTree

Because our method dose not base on alignment, it is impossible to perform the sampling-over-sites bootstrap or jackknife as in the standard methods. Our previous work has developed a bootstrap strategy which samples over proteins in the proteomes (see Ref. 13 in the main text for details). As a merit of this method, it can be used to test whether dropping or adding some genes will alter the tree topology. Our results show that the CVTree is quite robust to variations of gene-numbers within a considerable range ($\sim 30\%$).

The CVTree is robust to a certain degree to variations in genome annotation [1]

Although the distances between species are calculated based on their gene models, the tree topology is robust, to a certain degree, to variations of gene models used in tree construction. The above section has showed its robustness to the number of gene models. Moreover, it is quite stable to gene annotation versions as well.

We tested the topological stability of CVTree by using different versions of genome annotation to construct trees. The gene model of some fungal genomes have been changed rapidly because of lacking of evidences of transcripts and these changes have been reflected in different versions of genome annotation. We downloaded *M. grisea* proteome release 5 (released in 2007, denoted as M5 in this file) and 6 (2008, M6) as well as *F. graminearum* proteome release 1 (2004, F1) and 3 (2008, F3) from BROAD-FGI and FGDB (`http://mips.gsf.de/genre/proj/fusarium/`). There were 4 possible ways to get proteomes of the two species, i.e., (M5, F1), (M5, F3), (M6, F1) and (M6, F3). Figure 1 in the main manuscript was constructed using (M5, F3). By substituting (M5, F3) with the other three pairs, we built three trees. It turns out that the 4 trees have identical topology. In other words, using different gene models of the two species does not change the topology of CVTree.

[1] We thank the Second Reviewer for pointing out different annotation versions of *Maganporthe grisea* and *Fusarium graminearum* and providing the information of a second sequenced *A. niger* genome, i.e., *A. niger CBS513.88.*

However, we did find an example (*A. niger str. ATCC1015*) that different gene annotations of the same species led to two slightly different positions, but it did not affect other species in the tree. The directly downloaded *A. niger* proteome gave a tree that still placed it in the genus *Aspergillus*, but as sister taxon to the group consisting of *Aspergillus clavatus*, *Aspergillus fumigatus*, *Neosartorya fischeri*, *Aspergillus flavus*, *Aspergillus oryza* and *Aspergillus terreus* (Figure S3(a)). However, using the output of BGF (see Methods in the main text) to construct the 82-organism tree produced Figure 1. The same results were found when replacing *A. niger str. ATCC1015* by a newly sequenced *A. niger* genome (strain *CBS513.88*, which was not included in the 82 organisms because we did not know that it had been sequenced when this research was done). Similarly, when both strains were included in analysis, they were always placed as sister taxa that together constituted a clade showing the same pattern of movement with the change of genome annotation algorithms (Figure S3(a) and (b)). In each case, the placement of *A. niger* has rather high bootstrap support. Broader population sampling in this species and further analyses are needed to resolve the conflicts.

In short, CVTree is robust, to a certain degree, to variations of gene models. Not-very-bad annotations give reasonable trees.

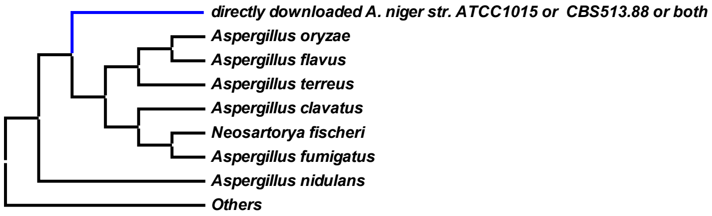

(a) Phylogeny resulted from directly downloaded *A. niger* proteomes. Both strains give identical topology. The BP values for ATCC1015, CBS513.88 and both are 93%, 100% and 100%, respectively.

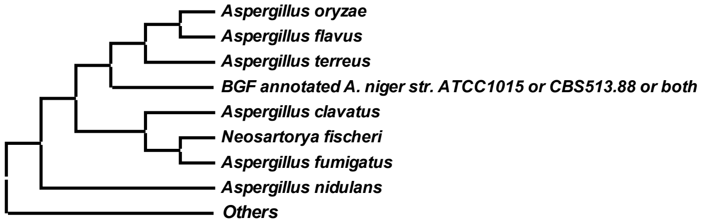

(b) Phylogeny resulted from BGF annotation of two *A. niger* strains. Both strains give identical topology. The BP values for ATCC1015, CBS513.88 and both are 85%, 100% and 100%, respectively.

Figure S3: Different genome annotation programs affect the placement of *A. niger*.
